# Comparison of Mechanical and Optical Properties of Multilayer Zirconia After High-Speed and Repeated Sintering

**DOI:** 10.3390/ma18071493

**Published:** 2025-03-27

**Authors:** Neslihan Güntekin, Burcu Kızılırmak, Ali Rıza Tunçdemir

**Affiliations:** Department of Prosthodontics, Faculty of Dentistry, Necmettin Erbakan University, 42090 Konya, Turkey; neslihanvarolnv94@gmail.com (N.G.); alirizatuncdemir@gmail.com (A.R.T.)

**Keywords:** biaxial flexural strength, multilayer, repeated sintering, speed sintering, translucency

## Abstract

This study aims to investigate the mechanical and optical properties of two different multilayer monolithic zirconia materials after the high-speed and repeated sintering process recommended by the manufacturers. In this study, specimens with a diameter of 12 mm and a thickness of 1 mm were fabricated using KATANA Zirconia YML (Kuraray Noritake) and IPS e.max ZirCAD Prime (Ivoclar Vivadent) multilayer zirconia. These specimens were processed with two different protocols to be used in the sintering process: high-speed and conventional sintering. Both protocols were repeated three times, after which the changes in the mechanical, microstructural and optical properties of the specimens were compared and analyzed. According to the biaxial flexural strength result, KATANA Zirconia YML (840.84 MPa) showed higher biaxial flexural strength compared to IPS e.max ZirCAD Prime (627.64 MPa) after repeated high-speed sintering. When the optical properties were analyzed, the translucency parameters of the IPS e.max ZirCAD Prime block were reliable in certain protocols. A comparison of mechanical and optical properties after repeated and high-speed sintering reveals that both materials offer advantages for different application requirements. The high biaxial flexural strength of KATANA Zirconia YML is more suitable for applications requiring strength. The homogeneous translucency of IPS e.max ZirCAD Prime is esthetically and optically safer for high-speed and repeated sintering processes.

## 1. Introduction

Nowadays, with the increasing esthetic expectations of people, ceramic restorations have become a strong alternative to metal ceramic restorations due to their excellent biocompatibility and esthetic acceptability [1,2]. With the production of high-strength ceramics with advanced technology, it has been observed that monolithic restorations have a better clinical performance than veneer restorations in terms of technical complications. Good biocompatibility, esthetic properties, high strength, toughness and translucency have been the main factors for the recent use of monolithic zirconia ceramics in dentistry. However, the low light translucency is still an important disadvantage of these ceramics [3,4]. Although monolithic zirconia restorations have been developed with microstructure modifications, their translucency values are still lower than other ceramic restorations [5,6]. The two main classes of materials commonly used in dental restorations are ceramics and resin-based composites. Ceramics are divided into three main groups: feldspar, glass (lithium disilicate, leucite) and polycrystalline ceramics (zirconia). Zirconia is stronger than the others, but opaquer. Due to their high strength properties, zirconia has been added to glass ceramics to reinforce them. Because ceramics are more vulnerable under tension, their flexural strength is an important mechanical property to determine their resistance to repetitive masticatory forces in the mouth. This property allows us to obtain information about the clinical lifetime of materials used in dental restorations by measuring the maximum force they can withstand before fracture [7,8].

Zirconia is a polymorphous crystal that can be found in three different forms (monoclinic, tetragonal, cubic) and can transform with temperature. The monoclinic (m-ZrO_2_) phase is stable at room temperature but is not suitable for clinical use due to its low translucency. In order for the tetragonal (t-ZrO_2_) and cubic (c-ZrO_2_) phase to be stable at room temperature, ionic molecules with a larger structure than zirconia, such as yttria, are included. When the amount of yttria increases, the cubic phase and translucency increases, but increased yttria content is associated with lower strength [9,10].

In the development of monolithic zirconias, multilayer zirconias have been produced to take advantage of high translucency and strength. Strength-gradient zirconia with different phase composition and yttria content in the layers have been introduced. In these materials, 3Y-TZP is used in the body region of the block for high strength, while 4Y-PSZ and 5Y-PSZ are used in the incisal and occlusal region for better translucency properties [11,12,13].

Sintering, which can change the structural, mechanical and esthetic properties of zirconia, is one of the most important steps to maintain the strength and esthetics of zirconia. Sintering reduces grain gaps, increases material density, and enhances light transmission. Different sintering protocols are known to affect the translucency and mechanical strength of zirconia [13]. The effects of high-speed sintering on translucency and flexural strength are important for multilayer zirconia with different contents in each layer and need to be further investigated. Long sintering durations can improve flexural strength through grain size variation and phase transformation [6]. However, decreased sintering duration results in faster production, lower cost and shorter patient appointment times. Shorter sintering durations accelerate restorations, allowing for same-day modifications and fewer treatment appointments. Repeated firing is applied 2–5 times on average for color correction, glazing or porcelain addition in zirconia. It has been reported that zirconia subjected to multiple firing cycles can increase bond strength, change flexural strength and microstructure [14,15]. However, studies examining the effects of sintering duration and repeated sintering on multilayer zirconia are insufficient.

The aim of this in vitro study was to investigate the effect of different and repeated sintering protocols on the phase composition, translucency and biaxial flexural strength of multilayer monolithic zirconia blocks. The null hypotheses are that high-speed, conventional and repeated sintering has no effect on the biaxial flexural strength of multilayer monolithic zirconia blocks (H1); high-speed, conventional and repeated sintering has no effect on the phase composition (H2) of multilayer monolithic zirconia blocks; and high-speed, conventional and repeated sintering has no effect on the translucency (H3) of multilayer monolithic zirconia blocks.

## 2. Materials and Methods

### 2.1. Specimen Preparation

**Figure 1:**
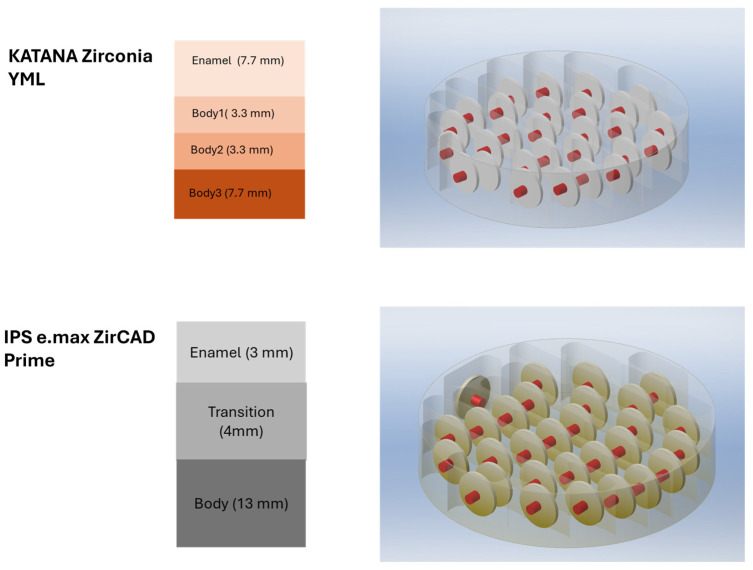
The placement of the designs in the blocks.

The multilayer zirconia blocks Katana Zirconia YML (Kuraray Noritake, Tokyo, Japan) and IPS e.max ZirCAD Prime (Ivoclar Vivadent, Schaan, Liechtenstein) were used to obtain specimens. Statistical power analysis G Power program (G*Power 3.1 software; Heinrich Heine University, Düsseldorf, Germany) was used to determine the number of specimens and according to the power analysis results, the number of specimens was determined as 64 (n = 16) with a margin of error of 5%, a sensitivity of 0.4 and a power of 85%. The specimen design was designed in SolidWorks (Dassault Systèmes SolidWorks Corp., Waltham, MA, USA, Solidworks 2021), with a sintered size of 12 mm diameter and 1 mm thickness and the zirconia blocks were milled on a milling machine (Coritec 350I, imes-icore GmbH, Stutensee, Germany). The placement of the designs in the blocks is shown in Figure 1.

**Figure 2:**
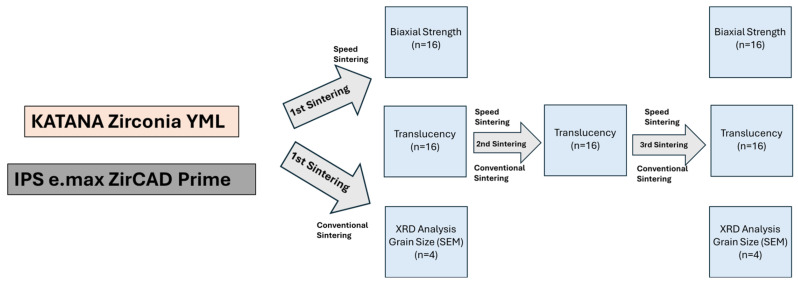
Flow chart of the experimental setup.

The milled specimens were sintered in a sinter furnace (HTS-2/M/ZIRKON-120, Zirkon Zahn, Kempten, Germany) in speed sintering (SS) and conventional sintering (CS) mode for YML and in speed sintering and conventional sintering mode for Prime according to the manufacturer’s recommended instructions, shown in Table 1. The flowchart of the experimental setup is shown in Figure 2.

### 2.2. Biaxial Flexural Strength

Biaxial flexural strength for the YML and Prime groups examined using a universal testing machine (Besmak Ltd., Ankara, Turkey) and the maximum force was recorded. Biaxial flexural strength calculations (MPa) were performed using a Poisson’s ratio of 0.25 and a piston tip diameter of 1 mm, loaded at a crosshead speed of 0.5 mm/min until fracture. The specimens were stored in 37 °C distilled water for 24 h before testing [16].

### 2.3. SEM Microstructural Analysis

One specimen was randomly selected from each subgroup and fixed to a plate with carbon tape. The specimens were coated with gold in a vacuum coating device (High Vacuum Sputter Coater–DST1-170, Kaiserslautern, Germany). The specimens were then placed in an SEM machine (Hitachi VP-SEM SU1510, Tokyo, Japan) and the center of the specimens was imaged at 10,000× magnification (2.00 kV). After the third sintering process, SEM imaging was repeated on the same specimens. For particle size analysis, Fiji-ImageJ software (National Institutes of Health, Bethesda, MD, USA, Fiji 2.1.0) was used and particle size analysis was completed using the linear cross-section method.

### 2.4. XRD Phase Analysis

After the first sintering, randomly selected specimens from each subgroup were used for X-ray diffraction analysis (XRD). For phase determination, Cu Kα (40 kV, 40 mA) was measured with an XRD device (Empyrean, Malvern Panalytical) at an angle of 20–40 degrees. After the third sintering process, XRD analysis was performed again on the same specimens. Rietveld analysis was performed using HighScore Plus software (Malvern Panalytical B.V., Almelo, The Netherlands, HighScore Plus 5.2) to evaluate the zirconia-phase content. The content information for Rietveld analysis was based on XRF values from the literature [17].

### 2.5. Translucency

Color measurement standards, color and spectral distribution were based on the International Commission on Illumination (CIE) standards. Calibration of the spectrophotometer was performed on a white and black background provided by the manufacturer. After the first sintering process was completed, all specimens were measured for L*, a* and b* values on black and white backgrounds using a spectrophotometer (Spectrophotometer PCE-CSM, PCE Instruments, Meschede, Germany) in the wavelength range of 400 and 500 nanometers. Measurements were made from the center of the specimens. The translucency parameter was determined based on the difference in measurements on black and white backgrounds and calculated according to the CIEDE2000 (TP00) formula shown below [6,18].

$$TP00=\sqrt{{\left(\frac{\Delta L^{\prime }}{K_{L}S_{L}}\right)}^{2}+{\left(\frac{\Delta C^{\prime }}{K_{C}S_{C}}\right)}^{2\;\;}+{\left(\frac{\Delta H^{\prime }}{K_{H}S_{H}}\right)}^{2}+R_{T}\;{\left(\frac{\Delta C^{\prime }}{K_{C}S_{C}}\right)}^{2\;\;}{\left(\frac{\Delta H^{\prime }}{K_{H}S_{H}}\right)}^{2}}\;$$

In this context, L′ denotes luminance, C′ chroma and H′ color, while RT denotes the interaction of the differences between C′ and H′ (blue region). SL, SC and SH were used to adjust the total color difference depending on the position of the color difference sample in L*, a* and b* coordinates on white (w) and black (B) backgrounds. KL, KC and KH represent correction terms [6]. According to the sintering protocols shown in Table 1, the second and third sintering processes were repeated and L*, a*, b* values were measured and recorded under the same conditions after each sintering and the translucency values were calculated.

### 2.6. Statistical Analysis

Data were analyzed with Minitab V14 and Jamovi V2.3.28. The conformity of the data to normal distribution was examined by Shapiro–Wilk test. A generalized linear model was used to compare the values conforming to normal distribution according to brand, sintering protocol and repeated sintering process and multiple comparisons were made with Tukey’s test. Robust ANOVA using the Wallrus package was used to compare values that did not fit the normal distribution, and multiple comparisons were made with the Bonferroni test.

## 3. Results

### 3.1. Biaxial Flexural Strength

Significant differences were observed when the biaxial flexural strength data of the materials were examined. When the variables of brand, sintering mode, repeated sintering are examined, there is a statistically significant effect (*p* < 0.001). The interaction between brand–repeated sintering and brand–sintering mode is statistically significant (*p* < 0.005). The triple interaction between brand, sintering mode and repeated sintering is not statistically significant (*p* = 0.661).

The biaxial flexural strength data of the groups are shown in Table 2. The mean fracture value of Prime after the 1st sintering process was 628.29 MPa, while the mean fracture value after the third sintering process was recorded as 626.98 Mpa. In YML, these values are 826.37 and 855.31 MPa, respectively. Contrary to these significant results, there was no significant effect of sintering mode–repeated sintering interaction on the fracture values (*p* = 0.727).

### 3.2. SEM Microstructural Analysis

**Figure 3:**
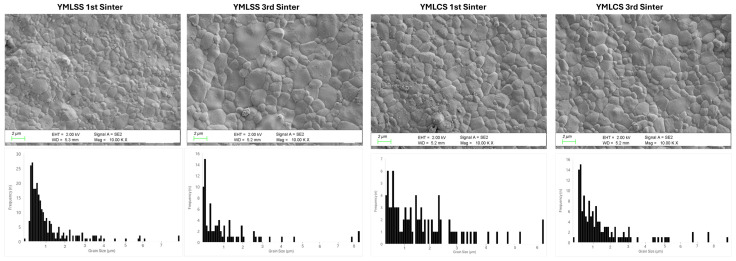
Representative YML SEM images and grain size distributions of the groups.

**Figure 4:**
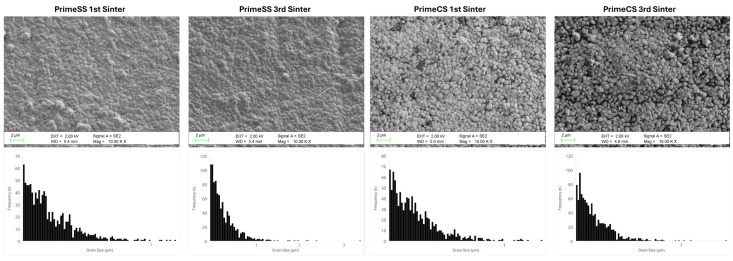
Representative Prime SEM images and grain size distributions of the groups.

In general, Prime specimens showed a smaller grain size and a narrower grain size distribution compared to YML specimens. The grain size distributions and SEM photomicrographs are shown in Figure 3 and Figure 4. With repeated sintering, the grain size distribution of Prime decreased significantly compared to YML and gaps started to be observed in the structure.

### 3.3. XRD Phase Analysis

**Figure 5:**
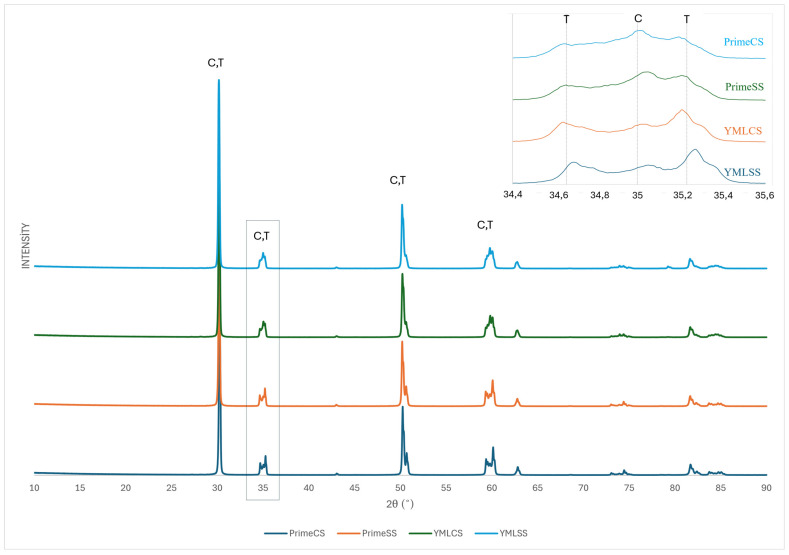
Representative XRD patterns of the 1st sintering (full 2θ range) and expanded view of the 34.4–35.6 (2θ).

**Figure 6:**
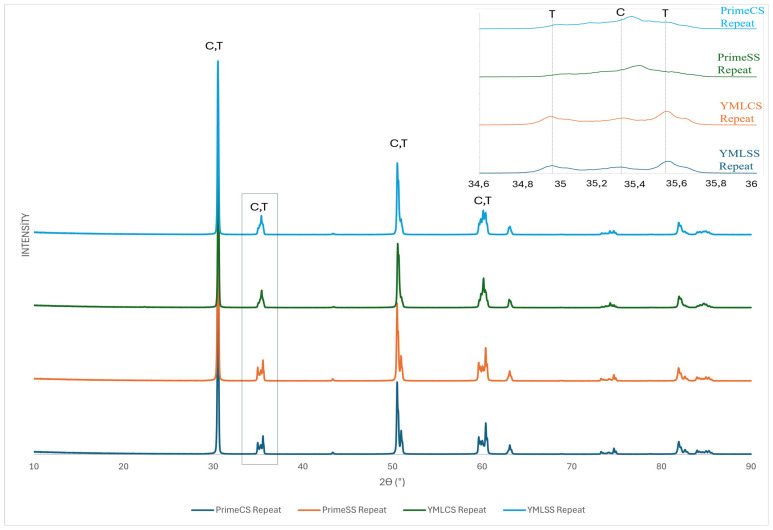
Representative XRD patterns of the 3rd sintering (full 2θ range) and expanded view of the 34.6–36 (2θ).

Rietveld analysis results of XRD data are shown in Table 3. The cubic phase was observed more in the high-speed sintered specimens than in the conventional sintered specimens. The cubic phase increased with repeated sintering and the difference is more pronounced in YML groups. XRD Diffractograms of the subgroups are shown in Figure 5 and Figure 6. As a result of repeated sintering, the similarity of the phase composition of the groups sintered according to the high-speed and conventional protocol increased.

### 3.4. Translucency

Sintering mode and repeated sinter values are presented in Table 4. It was observed that for the materials used, sintering modes and repeated sintering did not cause a significant difference in the translucency values for the specimens. According to the three-way ANOVA analysis, no significant differences were observed in two-way interactions with the main effects of brand, sintering mode and repeated sintering. However, significant differences were observed due to three-way interactions (*p* = 0.047). PrimeCS mean translucency after the second sintering is significantly higher than YMLSS and PrimeCS.

## 4. Discussion

According to the results of this in vitro study, high-speed, conventional and repeated sintering had significant effects on the biaxial flexural strength and phase changes in multilayer monolithic zirconia blocks and H1 and H2 were accepted, while no significant results were observed in the translucency parameters and H3 was rejected.

Increasing cubic phase ratio is related to the yttria content and as this ratio increases, the material might experience decreased mechanical strength [10,19]. However, according to the results of this study, although the cubic phase ratio of YML increased with repeated sintering, its strength was not adversely affected. Stawarczyk et al. reported that the highest biaxial flexural strength was obtained at sintering temperatures between 1400 °C and 1550 °C, while temperatures above 1600 °C changed the microstructure and reduced the strength [20]. The increase in the biaxial fracture strength of YML after the third sintering process can be explained by having the highest sintering temperature protocol (1550–1560 °C). The material structure became more compact during repeated sintering. According to Griffith’s law, the fracture strength of a material is related to the grain size and crack size in its internal structure, and materials with smaller defects exhibit higher fracture strength. The high fracture strength of YML, which exhibits a more homogeneous structure with repeated sintering, despite the high c-ZrO_2_ content and large grain structure, can be attributed to its increased structural accuracy [17,21].

From the enamel to the lowest layer, yttrium content is known to vary from 5% to 3 mol% Y2O3 in Prime and from 5% to 4 mol% Y2O3 in YML. This result is due to the changes in yttrium content due to the phase content of c-ZrO_2_ and t-ZrO_2_ [10,17]. During the sectioning process of the specimens, it was found that the YML block was taken from the body 2 and body 3 layers, while the Prime block was taken from the transition layer with a higher rate. In a study comparing the biaxial strength of YML and Prime, it was reported that the strength of the body 2 and 3 layers of YML was higher than the transition layer of Prime; this difference was related to yttrium content and grain size [17]. The high biaxial flexural strength of YML can be explained by this situation. There was no change in the biaxial flexural strength of Prime after repeated sintering. This result is in line with studies indicating that multiple sintering processes do not affect the biaxial flexural strength of zirconia [21,22]. Increasing sintering temperatures are known to reduce porosity and lead to densification of the crystal structure of zirconia. This provides a basis to explain the lack of decrease or increase in strength after repeated and high-speed sintering [23].

It is stated in the literature that the particle size increases with the increase in Yitria content and that the strength decreases accordingly [3]. In microstructure examinations, it was observed that the particle size of YML was higher compared to Prime. It is thought that this situation could be due to differences in yttria content and changes in production processes. Additionally, it can be stated that the YML is characterized by a high particle content. Although the particle size is significantly higher than Prime, there are studies in the literature indicating that YML has higher flexural strength [10,17]. More studies are needed to investigate the microstructure of YML after repeated sintering.

In the literature, it has been reported that increasing the sintering duration and temperature affects the particle size in the direction of growth [1]. In studies comparing speed and conventional sintering modes, it was reported that there was no significant difference in the particle size of 3Y-TZP grade zirconia, but zirconia with high translucency exhibited different behavior. In these studies, it was stated that high-speed sintering method should be preferred for zirconia with high translucency to preserve the microstructure and prevent particle growth [6,24].

Similarly to these studies, it was observed in Prime that the grain structure was preserved during high-speed sintering, while the gaps were formed in the microstructure as a result of conventional and repeated sintering. This may be due to structural changes due to the sintering process. In the study by Shin et al. on Katana UTLM, no significant change in particle size was observed after high-speed sintering [25]. Similarly, it was concluded that prolonged sintering duration with high-speed and repeated sintering in YML blocks preserved the particle structure and the stable structure was maintained if appropriate sintering protocols were followed.

The increase in Al_2_O_3_ content leads to a larger grain structure. However, although Prime blocks have higher Al_2_O_3_ content in each layer compared to YML, they show lower grain size [17]. While no change in particle size was observed in Prime blocks in conventional sintering mode as the sintering duration increased, it was reported that the homogeneous structure deteriorated. During the sintering process, Al_2_O_3_ is repositioned, resulting in uneven distribution and increased grain boundary structure [26]. It can be concluded that high Al_2_O_3_ content due to increased sinter duration due to repeated sintering leads to an increase in micro-gaps.

As the temperature increases, compacting occurs in the zirconia crystal structure, while porosity and defects decrease. Porosity has a significant effect on the translucency properties of zirconia, which directly affects the optical performance of the material [3]. However, it is considered a contradiction that the translucency and strength of the Prime block do not show significant differences with sintering mode and repeated sintering. According to the information provided by the manufacturers, YML consists of four layers (enamel, body 1, body 2 and body 3), while Prime consists of three layers (enamel, transition, body). This is thought to be due to the fact that SEM imaging was only taken from one region, the central part of the specimens. Furthermore, the variability of the yttria content of the interlayer may adversely affect structural stability. However, strength was compensated by the fact that the specimen contents consisted of different layers. The particle structure of all layers of Prime with repeated sintering should be investigated in future research.

According to the results of this study, it was observed that the phase ratio of c-ZrO_2_ increased as a result of repeated sintering. It was observed that phase change occurred in zirconia with 3 Y-TZP content because of high-speed sintering, while 5 Y-TZP zirconia was not affected by high-speed sintering and was more stable due to the higher yttrium causing rapid diffusion [24]. Repeated sintering is thought to produce similar effects with high-speed sintering. In both Prime and YML, increasing the ratio of 3 Y-TZP from the enamel layer to the body caused more phase change [17].

In studies where the effects of sintering temperature and duration were evaluated, there are studies that examined the effect of particle size increase on translucency and reported different results [1,6]. It is known that coloring pigments added to zirconia reduce the translucency [27]. It has been reported that tetragonal zirconia polycrystals stabilized with dyed yttrium containing Fe_2_O_3_ exhibit a translucency similar to unpainted zirconia ceramics; however, colored zirconia ceramics show lower translucency. It was also stated that zirconia with large grains offer high translucency due to their narrow grain boundaries and that the effect of c-ZrO_2_ content on translucency is greater than that of grain size [23].

In this study, although YML has a larger particle structure and a significant increase in cubic phase content was observed as a result of high-speed and repeated sintering, no statistically significant difference was detected in terms of translucency with Prime blocks. This can be interpreted as suggesting that additional pigmentations in YML blocks may prevent the formation of significant differences after sintering, according to the information provided by the manufacturer [17,28].

As previously mentioned, YML and Prime are materials with different layer contents and thickness, as outlined in Figure 1. In a study examining the layer properties of YML and Prime blocks, the translucency of the “enamel” layer of YML was found to be higher than that of the “body 2” and “body 3” layers, but no significant difference was detected between the “body” layers. In Prime blocks, the translucency of the “enamel” layer was higher than that of YML, while the translucency of the “body” layers of YML was higher than that of Prime [17]. Based on this information, it could be concluded that the standard specimens obtained from these two materials with different layer content and thickness offer similar light transmittance and clinically comparable results.

Repeated sintering had no significant effect on the translucency (*p* = 0.289). This indicates that the optical properties of the materials remain stable when the sintering protocols recommended by the manufacturers are followed. However, a significant difference (*p* = 0.047) was found in the interaction of material, sintering mode and repeated sintering process. This indicates that some specific combinations could produce different results. It was observed that after the second sintering process in Prime, conventional sintering showed significantly higher translucency than the first high-speed sintering and the highest translucency was observed in this group. It has been reported that rapid temperature increases and shortened sintering durations may increase the compacted particle sizes, resulting in lower translucency. Therefore, it can be considered as a possible consequence of the conventional sintering protocol leading to higher translucency values in the second sintering [26].

The in vitro design used in this study does not accurately reflect the complex and variable conditions of clinical practice. The effects of occlusal loads and prolonged thermal cycling on material performance were not evaluated. Additionally, SEM analyses were only taken from specific regions, which may have limited the full representation of heterogeneities in phase structure and microstructure for all specimens. In future studies, it is recommended to conduct detailed analyses from different layers with larger specimen sizes, as well as long-term clinical simulations.

## 5. Conclusions

In this study, the effects of sintering mode and repeated sintering processes on the translucency, mechanical strength and microstructural properties of different multilayer zirconia blocks were investigated. The findings showed that different sintering protocols gave generally stable results for both materials. Katana YML was characterized by higher biaxial flexural strength values in the high-speed sintering mode, while the Prime block showed a more homogeneous performance in terms of translucency. In clinical practice, the material and sintering protocol should be determined according to the esthetic and mechanical requirements of the restoration. Future research is needed to evaluate the long-term effects of repeated sintering on zirconia restorations in vivo.

## Figures and Tables

**Table 1:** The sintering protocol of YML and Prime.

	YMLSS	YMLCS	PrimeSS	PrimeCS
Heating Rate (°C/min)	50 °C/min (1400 °C) 4 °C/min (1500 °C) 10 °C/min (1560 °C)	10 °C/min (1550 °C)	60 °C/min (1000 °C) 3 °C/min (1530 °C)	10 °C/min (900 °C) 3.3 °C/min (1500 °C)
Sintering	1560 °C for 16 min	1550 °C for120 min	1000 °C for 10 min 1530 °C for 60 min	900 °C for 30 min 1500 °C for 120 min
Cooling Rate (°C/min)	50 °C/min (800 °C)	10 °C/min (37 °C)	50 °C/min (1100 °C) 60 °C/min (100 °C)	10 °C/min (900 °C) 8.3 °C/min (300 °C)

**Table 2:** Comparison results of biaxial flexural strength data according to brand, sintering mode (CS-SS) and repeated sintering (MPa).

Sintering Mode	Repeated Sintering	Brand		Mean
		Prime	YML	
SS	1st sintering	631.47 ± 8.72	835.29 ± 9.06	733.38 ± 104.01
	3rd sintering	629.86 ± 26.12	866.95 ± 24.85	748.41 ± 123.15
	Mean	630.67 ± 19.15 ^C^	851.12 ± 24.43 ^A^	740.89 ± 113.27
CS	1st sintering	625.10 ± 5.8	817.46 ± 10.86	721.28 ± 98.2
	3rd sintering	624.11 ± 10.38	843.67 ± 33.85	733.89 ± 114.33
	Mean	624.61 ± 8.28 ^C^	830.56 ± 28.06 ^B^	727.59 ± 105.85
Mean	1st sintering	628.29 ± 7.97 ^C^	826.37 ± 13.36 ^B^	727.33 ± 100.47
	3rd sintering	626.98 ± 19.74 ^C^	855.31 ± 31.49 ^A^	741.15 ± 118.03
	Mean	627.64 ± 14.94	840.84 ± 28.07	734.24 ± 109.37

A–C: No difference between interactions with the same letter.

**Table 3:** XRD-Rietveld analysis data (wt%).

	t-ZrO_2_ (wt%)	m-ZrO_2_ (wt%)	c-ZrO_2_ (wt%)
YMLCC	68.5	0.4	31.1
YMLSC	66		34
PrimeCS	71		29
PrimeSS	68.6		31.4
YMLCC 3rd	62.5		37.5
YMLSC 3rd	60.2		39.8
PrimeCS 3rd	67.3		32.7
PrimeSS 3rd	60.8		33.1

**Table 4:** Results of multiple comparisons of translucency means by brand, sintering mode (CS-SS) and repeated sintering.

Repeated Sintering	Mode	Brand		Mean
		YML	Prime	
1st sintering	SS	7.34 ± 0.12 ^A^	7.51 ± 0.08 ^AB^	7.46 ± 0.05
	CS	7.66 ± 0.13 ^ABC^	7.98 ± 0.61 ^ABC^	7.58 ± 0.08
	Mean	7.52 ± 0.08	7.49 ± 0.06	7.51 ± 0.04
2nd sintering	SS	7.6 ± 0.16 ^ABC^	7.7 ± 0.04 ^ABC^	7.69 ± 0.05
	CS	7.75 ± 0.12 ^ABC^	8.04 ± 0.11 ^C^	7.92 ± 0.07
	Mean	7.72 ± 0.08	7.88 ± 0.06	7.81 ± 0.05
3rd sintering	SS	7.5 ± 0.14 ^ABC^	7.83 ± 0.08 ^BC^	7.71 ± 0.05
	CS	7.77 ± 0.13 ^ABC^	7.55 ± 0.11 ^ABC^	7.68 ± 0.08
	Mean	7.67 ± 0.08	7.7 ± 0.06	7.7 ± 0.05
Mean	SS	7.52 ± 0.07	7.68 ± 0.04	7.62 ± 0.03
	CS	7.75 ± 0.07	7.7 ± 0.07	7.73 ± 0.05
	Mean	7.64 ± 0.05	7.69 ± 0.04	

A–C: No difference between interactions with the same letter.

## Data Availability

The original contributions presented in this study are included in the article. Further inquiries can be directed to the corresponding author.
